# P-2071. Reaching the Hard-to-Reach via the ACCELERATE Model of HIV Care: Telehealth, Rapid Restart and Follow-up

**DOI:** 10.1093/ofid/ofaf695.2235

**Published:** 2026-01-11

**Authors:** Hilal Abdessamad, Thomas T Deem, Blair Thedinger, Wassim Jamaleddine, Anis Buttar-Miller, Ransome Drexler, Dima Dandachi

**Affiliations:** University of Missouri, Columbia, Missouri; Gilead Sciences, Inc., Foster City, California; KC CARE Health Center, Kansas City, Missouri; Central Michigan University, Columbia, Missouri; University of Missouri, Columbia, Missouri; University of Missouri, Columbia, Missouri; University of Missouri - Columbia, Columbia, MO

## Abstract

**Background:**

Patient retention in HIV care is key for optimal health outcomes and Ending the HIV Epidemic (EHE) in the United States. Missouri (MO) is a Phase I jurisdiction due to high rural HIV transmission. ACCELERATE is an on-going implementation study across MO that assesses the effectiveness of an intervention to identify and rapidly relink people with HIV (PWH) who are out of care (OOC).

**Figure 1:**
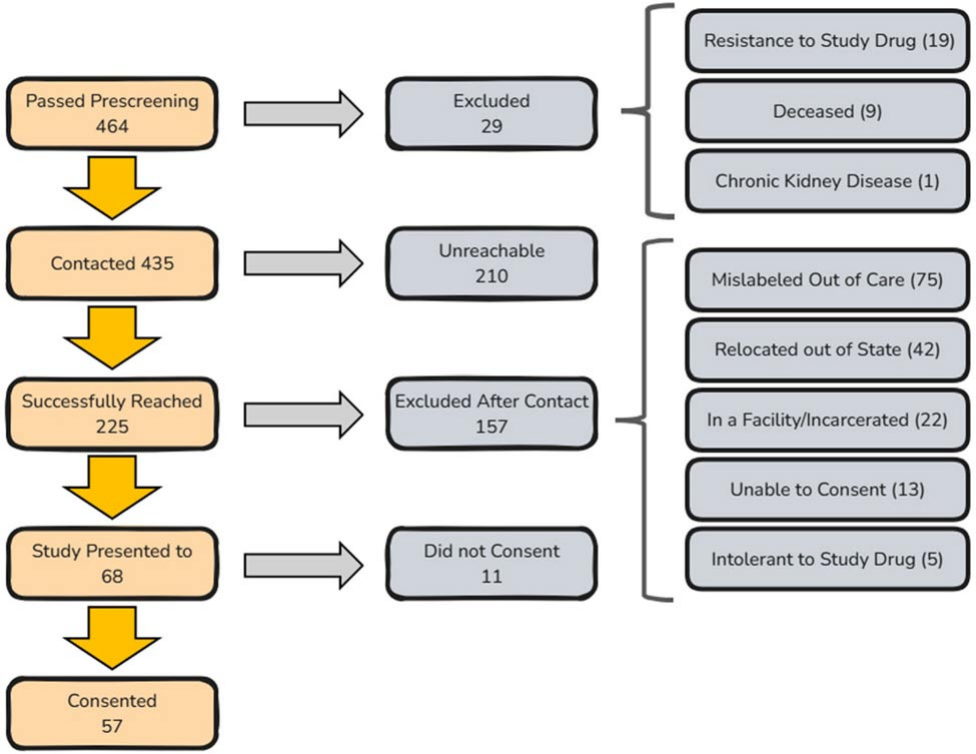
Outreach Efforts in ACCELERATE Flowchart from Prescreening of OOC PWH until Study Enrollment. Unreachable: PWH who could not be contacted after sharing study flyer, 4 phone call across 3 weeks, a patient portal message and an email. Out of State: Moved outside Missouri, one of the exclusion criteria of ACCELERATE. Unable to Consent: People who cannot understand and sign the informed consent. Study Drug: Bictegravir/Emtricitabine/Tenofovir Alafenamide

**Figure 2:**
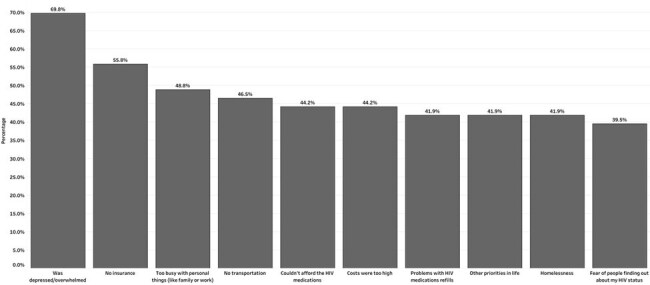
Top 10 Barriers to HIV Care Selected by PWH who were OOC (%, N=43) As part of the baseline survey, respondents chose which barriers were important reasons to fall out of care. For each barrier, the percentage of respondents who identified it as "important" was calculated. The top 10 barriers were represented by this figure.

**Methods:**

We engaged adult OOC PWH in 4 clinics across MO through a data to care analysis and enhanced contact algorithm. OOC was defined as a lack of medical visit in the last 6 months and off antiretroviral therapy (ART) for at least 1 month. Prior to an in-person visit, individuals participated in a telehealth (TLH) provider appointment that included rapid start of a 30-day supply of study-provided Bictegravir/ Tenofovir Alafenamide/ Emtricitabine without baseline labs. PWH completed surveys at baseline and after the 1 month clinic visit.

**Table 1:**
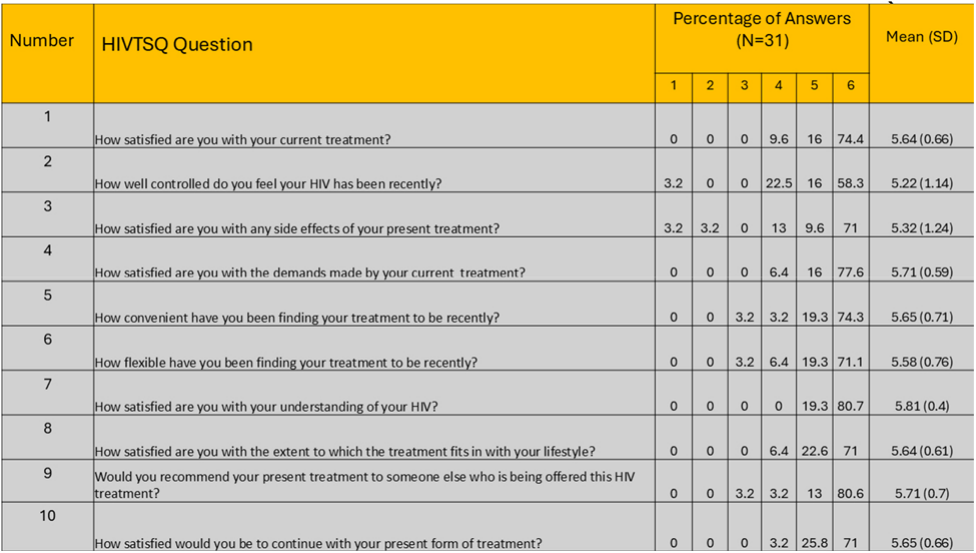
Ten-item HIV Treatment Satisfaction Questionnaire (HIVTSQ) Findings

The responses to the HIVTSQ questionnaire are scaled from 1 to 6, where 1 represents the least favorable response and 6 represents the most favorable one.

**Results:**

Phone calls were the most successful contact method. We reached 225/464 (49%) PWH, 150/225 (63%) confirmed OOC, 68/150 (45%) were eligible, and 57/68 (84%) were enrolled (Figure 1). On average, participants have been off ART for 15 months (SD 22). Participants had a mean age of 42 (SD 11), were 80% male and 36% black. At baseline, 52% reported substance use, 48% had an Internalized AIDS Stigma Scale score of 5+, 62% screened positive for anxiety with the Generalized Anxiety Disorder-2 survey and 60% for depression with the Patient Health Questionnaire-2 survey. Among barriers to care, mental health causes ranked highest, followed by financial concerns (Figure 2).

Using the Customer Assessment of Health Care Providers and Systems survey, 33/43 (77%) of PWH rated their TLH visit of 8 or above (scale 1 - 10). Of PWH who reached the 1 month follow-up (n=41), 80% attended their clinic visit, 30/31 (97%) reported missing less than 5 doses in 30 days, and most reported satisfaction with their ART using the HIV Treatment Satisfaction Questionnaire (Table 1).

**Conclusion:**

Despite the high prevalence of financial difficulties, mental health challenges and substance use among participants, ACCELERATE was acceptable to PWH. Utilizing an integrated, rapid-start telehealth intervention shows promise as a re-engagement strategy to accelerate our efforts towards achieving the targets of EHE in the US.

**Disclosures:**

Thomas T. Deem, MSN, FNP-BC, AAHIVS, Gilead Sciences, Inc.: Employee Compensation|Gilead Sciences, Inc.: Stocks/Bonds (Private Company) Blair Thedinger, MD, Gilead: Advisor/Consultant|Viiv: Honoraria Dima Dandachi, MD, MPH, Gilead Sciences: Grant/Research Support|Gilead Sciences: Honoraria|ViiV Healthcare: Grant/Research Support|ViiV Healthcare: Honoraria

